# Quality measures for the care of patients with lateral epicondylalgia

**DOI:** 10.1186/1471-2474-14-310

**Published:** 2013-10-30

**Authors:** Francisco Minaya-Muñoz, Francesc Medina-Mirapeix, Fermin Valera-Garrido

**Affiliations:** 1 MVClinic. Juan Antonio Samaranch Torelló St., 6B. Fitness Sports Center Valle de Las Cañas, 28223, Pozuelo de Alarcón, Madrid, Spain; 2 Faculty of Medicine, San Pablo CEU University, Madrid, Spain; 3 Fremap Hospital, Majadahonda, Madrid, Spain; 4 Department of Physiotherapy, University of Murcia, Campus Mare Nostrum, Universidad de Murcia, 30100, Campus de Espinardo Murcia, Spain

**Keywords:** Lateral epicondylalgia, Quality of care, Recommendations, Quality measures, Clinical quality indicators

## Abstract

**Background:**

Lateral epicondylalgia (LE) defines a condition of varying degrees of pain near the lateral epicondyle. Studies on the management of LE indicated unexplained variations in the use of pharmacologic, non-pharmacological and surgical treatments.

The main aim of this paper was to develop and evaluate clinical quality measures (QMs) or quality indicators, which may be used to assess the quality of the processes of examination, education and treatment of patients with LE.

**Methods:**

Different QMs were developed by a multidisciplinary group of experts in Quality Management of Health Services during a period of one year. The process was based following a 3-step model: i) review and proportion of existing evidence-based recommendations; ii) review and development of quality measures; iii) pilot testing of feasibility and reliability of the indicators leading to a final consensus by the whole panel.

**Results:**

Overall, a set of 12 potential indicators related to medical and physical therapy assessment and treatment were developed to measure the performance of LE care. Different systematic reviews and randomized control trials supported each of the indicators judged to be valid during the expert panel process. Application of the new indicator set was found to be feasible; only the measurement of two quality measures had light barriers. Reliability was mostly excellent (*Kappa* > 0.8).

**Conclusions:**

A set of good practice indicators has been built and pilot tested as feasible and reliable. The chosen 3-step standardized evidence-based process ensures maximum clarity, acceptance and sustainability of the developed indicators.

## Background

Lateral epicondylalgia (LE), also known as tennis elbow or lateral epicondylitis, defines a condition of varying degrees of pain or point tenderness on or near the lateral epicondyle. Functional use of the involved upper extremity, especially during gripping activities usually exacerbates pain symptoms [1,2]. Although LE has been traditionally defined as an inflammatory process, several more recent studies have shown that the pathophysiology of this process is degenerative in nature [3-5]. Regardless of the aetiology, LE represents a pathology that accounts for lost recreation time, decreased quality of life, and work-related disability claims, and LE also has widespread social, financial, and clinical implications [6].

In the past, studies on the management of LE indicated substantial and unexplained variations in the use of pharmacologic, non-pharmacological and surgical treatments [7,8]. Currently, there is convincing evidence demonstrating that multifactorial intervention programs involving a multidisciplinary team are effective in reducing both pain and disability of patients with lateral epicondylalgia [7,9]. However, little is still known about the degree of implementation of best practices recommendations for these patients. This study was designed to address this void of knowledge by developing quality measures (QMs) for LE care [10].

QMs, or quality indicators, enable the user to quantify the quality of a selected aspect of care by comparing it to an evidence-based criterion that specifies quality [11]. Thus, QMs can measure the performance of an individual facility over time, compare the degree of implementation of the best practices care between different health care providers, and identify areas for improvement [12]. Methodological approaches to QMs’ development have been described. Important attributes of high quality QMs are their validity, their feasibility and their reliability. Furthermore, they need to be easily understandable for providers and achievable [13,14].

The objective of this paper was to develop a set of valid and understandable clinical QMs that may be used to assess the degree of implementation of the best processes of examination, education and treatment of patients with LE, and to pilot test their feasibility and reliability.

## Methods

For the development of these evidence-based quality indicators, we assembled a multidisciplinary panel of recognized experts from the disciplines of traumatology, nursing, physical therapy and psychopedagogy. The study design and methods were reviewed and approved by the Academic Review Committee of Quality Management Program at the Faculty of Medicine of University of Murcia (Murcia, Spain).

Research into quality of care, together with epidemiological expertise, helped ensure methodological integrity of the clinical indicators and the ensuing sound approach to data collection and data analysis. The 6-member panel included people from geographically diverse regions including rural and urban areas.

**Figure 1:**
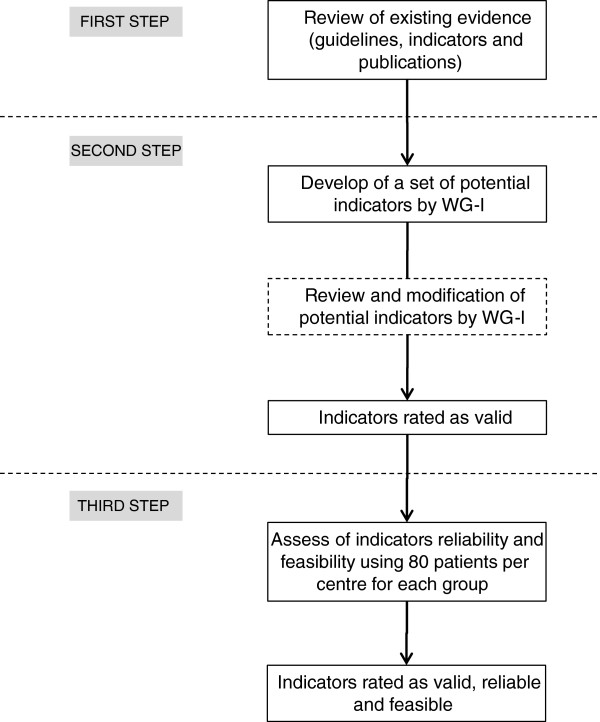
Diagram of the 3-step process developed to define the quality measures.

We used a 3-step process to define the QMs (Figure 1). First, we performed literature reviews to provide the existing evidence-based recommendations in relation to the three main components of LE care (examination, education and treatment). Secondly, we reviewed the existing QMs, identified evidence-based recommendations no covered by existing QMs and developed new ones to cover all evidence-based recommendations. Thirdly, we tested for feasibility and reliability of the accepted set of QMs.

### Review and proportion of existing evidence-based best recommendations

A working group of panel members performed a comprehensive literature search to identify basic systematic reviews (SRs) and clinical practice guidelines (CPG) that pertained to LE care. Literature searches of both reviews and guidelines were conducted in the following databases over the 10 previous years (2001–2011): Medline, Cochrane Library and Ovid, PEDro and ENFISPO. Additionally, searches of clinical guidelines were performed using the following specific guidelines databases: National Guideline Clearinghouse of AHRQ [11], the Scottish Intercollegiate Guidelines Network (SIGN) [15], the New Zealand Guidelines Group (NZGG) [16], National Institute for Health and Clinical Excellence (NICE) [17], and GuiaSalud [18].

The working group retained recommendations which had supporting evidence level *A* (one or more randomized controlled trials, with or without systematic review) or *B* (significant observational studies), with strength of recommendations categorized as *strong* or *weak* according to specific classifications, also taking into account a positive balance between the desirable and undesirable consequences and the lower costs of the alternative management strategies [19]. To facilitate the next phase, we agreed to provide a summary of the recommendations stating the population to which these applied to and the process of care measured. Thus, when it was possible, we created a phrase using the structure IF-THEN.

### Review and development of quality indicators

Initially, the working group performed a comprehensive search of quality indicators in the United States’ National Quality Measures Clearinghouse of the AHRQ and in articles indexed in the following databases for the 10 previous years (2001–2011): Medline, Scopus and Psycinfo. For each of the existing indicators, the working group provided an objective and a summary of the available evidence, in order to assess whether to support or refute them.

The working group elaborated new clinical indicators or adapted existing ones when valid indicators were lacking for some of the recommendations selected during the first phase. Standardized reports of potential indicators were suggested by the working group to facilitate discussion within the whole panel before selection.

**Figure 2:**
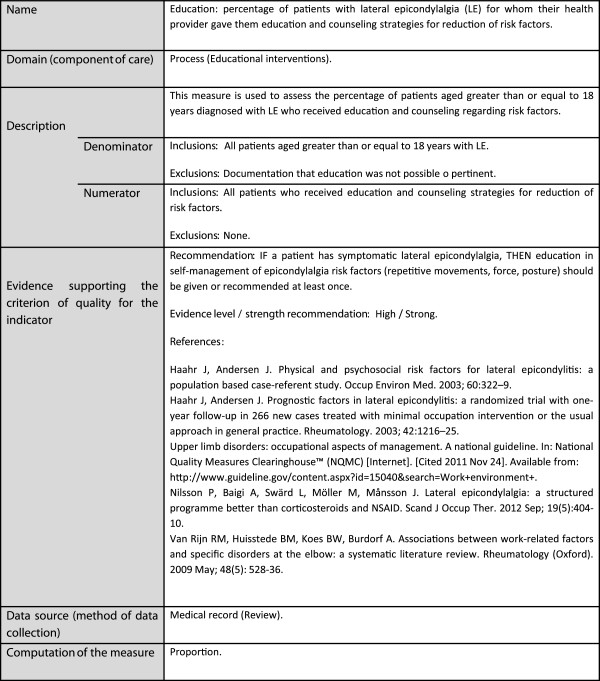
Exemplary presentation of standardized report for an evidence-based quality measure.

Following a proposal of the Agency for Healthcare Research and Quality (AHRQ) on measure attributes of the National Quality Measures Clearinghouse [11], the report included: name; domain (process, access) and component of health care quality to be covered (examination, educational or therapeutic interventions); description of the indicator, including definition of a suitable patient for whom the quality is valid (included and excluded population for numerator and denominator); evidence supporting the indicator, including recommendations, evidence level and strength of the recommendation and references (original publications, guidelines, indicators and consensus statements); data source and method of measurement (review of medical record audits); computation of the measure (rate or proportion). Figure 2 shows this standardized report as applied to an indicator.

The panel discussed the validity of each of the proposed measures and retained those with adequate evidence of the good practice measured and achievable or with the indicator compliance under control of providers.

### Pilot testing

The set of quality measures was them pilot tested by two members of the panel to check whether the measurement of performance of healthcare providers by the QMs was feasible and reliable on a routine basis. This study was conducted in three hospitals located in Barcelona, Vigo (Galicia) and Madrid. Each measure had its own eligibility criteria, depending on the type of patients it was aimed at. We selected only a randomized sample of 80 patients with LE who had received treatment during the 12 month time period before the testing date as we assumed that all QMs could have the minimal sample size of 15 patients [20]. The database of medical records belonging to each participant centre and diagnostic codes (CMBD) were used for identifying subjects and selecting the sample.

To test for feasibility of the QMs the two examiners reviewed databases and selected medical records, reporting when it was not possible to assess some QMs and related reasons. Predictable reasons encountered were: difficulty to identify cases or misreported information in medical records.

Intra-rater and inter-rater designs were used to examine the reliability of QMs. To test for intra-rater reliability, an examiner assessed each QM in two occasions spaced out over a period of 7 days. To test inter-rater reliability, an additional examiner carried out assessments using the same subjects’ medical records. Reliability indexes were determined through the calculation of the Kappa index using SPSS v.15. Alternatively, we used the general agreement percentage when occurrence or absence of evaluated processes of care was higher than 85%, because Kappa index could be biased. We interpreted Kappa levels using established conventions: values ranging from 0.40 to 0.59 may be considered moderate, 0.60 to 0.79 substantial, and ≥ 80 excellent [21]. When the values of the Kappa index were less than 0.60 or the values of the general agreement index where less than 0.95, the quality indicators were reviewed by the whole panel.

## Results

Table 1 includes the recommendations identified in the first phase, and their evidence level and strength. None were based on CPG. From these recommendations, a total of 12 potential QMs were created and accepted for the pilot testing: 3 were related to assessment interventions, 1 to educational interventions and 8 to therapeutic interventions (Table 2).

**Table 1:** Evidence-based recommendations for lateral epicondylalgia care

**Components of care**	**Evidence-based recommendations**	**Evidence level / strength recommendation**
I. Patient exam		
Physical Examination	1. IF a patient begins a treatment for lateral epicondylalgia, THEN evidence that the affected tendon was examined should be documented (at least orthopaedic tests).	B / Strong [22]
Pain and functional assessment	2. IF a patient has symptomatic lateral epicondylalgia, THEN pain should be assessed (at least the intensity level) upon initiation of a new treatment at least once.	B / Strong [23,39,43]
	3. IF a patient has symptomatic lateral epicondylalgia, THEN functional status should be assessed upon initiation of a new treatment at least once.	B / Strong [23,39,43]
II. Educational interventions		
Education	4. IF a patient has symptomatic lateral epicondylalgia, THEN education about self-management of risk factors (repetitive movements, etc.) should be given or recommended at least once.	A / Strong [28-30]
III. Therapeutic interventions		
1. Pharmacological therapy		
First line	5. IF a patient is started on pharmacological therapy to treat lateral epicondylalgia, THEN NSAIDs should be tried first.	B / Weak [31,32]
Prophylaxis	6. IF a patient with a risk factor for GI bleeding (age ≥75, peptic ulcer disease, history of GI bleeding) is treated with a NSAID, THEN he or she should be treated concomitantly with inhibitors (e.g. proton pump inhibitor, misoprostol, etc.).	A / Strong [35]
2. Physical therapy		
First line	7. IF a patient is started on physical therapy to treat lateral epicondylalgia, THEN a program of exercise therapy (training epicondyle muscles excentrically and concentrically) should be tried first.	A / Strong [24,25,36-38]
	8. IF a patient is started on physical therapy to treat lateral epicondylalgia, THEN manual therapy by mobilization with movement should be tried first.	A / Strong [26,39]
	9. IF a patient is started on physical therapy to treat lateral epicondylalgia, THEN laser therapy should be tried first.	A / Strong [40]
Time to referral	10. IF a patient is treated with corticosteroid injection for lateral epicondylalgia, THEN a multimodal program of physical therapy should be initiated early before 14 days.	A / Strong [43]
3. Other therapeutic interventions		
First line	11. IF a patient is treated with platelet-rich plasma for lateral epicondylalgia, THEN infiltration into the extensor digitorum communis tendon by peppering technique should be recommended.	B / Strong [42,44-46]
Surgery	12. IF a patient was symptomatic after a minimum of 6–12 months of conservative therapies, THEN surgery should be recommended.	A / Strong [47-49]

Evidence level *A* (one or more randomized controlled trials, with or without systematic review) and *B* (significant observational studies).

Strength of recommendations *strong* and *weak* according to specific classifications.

**Table 2:** Quality measures for lateral epicondylalgia care and their reliability

**Clinical quality measures**	**n**	**Kappa index**		**General agreement percentage (%)**	
		**Inter-rater**	**Intra-rater**	**Inter-rater**	**Intra-rater**
I. Patient exam					
1. Physical examination: percentage of patients with LE for whom the affected tendon was examined (at least orthopedic tests).	80	-	-	100	100
2. Pain assessment: percentage of patients with LE who had pain assessment upon initiation of a new treatment.	80	-	-	95	100
3. Functional assessment: percentage of patients with LE who had functional assessment upon initiation of a new treatment.	80	-	-	85	95
II. Educational interventions					
1. Education: percentage of patients with LE for whom their health provider gave them education and counselling for risk factor reduction strategies.	80	0.8	0.9	-	-
III. 1. Pharmacological therapy					
1. Pharmacological therapy for LE: percentage of patients who received pharmacological therapy for LE and who received NSAIDs as first line.	75	-	-	100	100
2. Prophylaxis for gastrointestinal bleeding: percentage of patients with NSAIDs for LE who concomitantly received inhibitors (e.g. proton pump inhibitor, misoprostol, etc.).	24	0.9	1	-	-
III. 2. Physical therapy					
1. Physical therapy for LE: percentage of patients who received physical therapy for LE and who received a program of exercise therapy as first line.	42	-	-	95	100
2. Physical therapy for LE: percentage of patients who received physical therapy for LE and who received manual therapy as first line.	42	0.9	1	-	-
3. Physical therapy for LE: percentage of patients who received physical therapy for LE and who received laser therapy as first line.	42	0.9	1	-	-
4. Time of referral: percentage of patients with LE who received corticosteroid injection and who initiated a multimodal program of physical therapy within 14 days of the injection.	17	1	1	-	-
III. 3. Other therapeutic interventions					
1. Platelet-rich plasma (PRP) therapy for LE: percentage of patients who received PRP therapy for LE and who received PRP therapy as first line (*).	1	N/A.	N/A.	N/A.	N/A.
2. Surgery intervention for LE: percentage of patients who were symptomatic after a minimum of 6–12 months of conservative therapies and who underwent surgery (*).	14	N/A.	N/A.	N/A.	N/A.

N/A. Not applicable.

-: Index not calculated. The general agreement percentage was used when occurrence or absence of evaluated processes of care was higher than 85%.

*.QMs in the text which had slight feasibility problems.

### Feasibility and reliability

In the pilot testing, items with feasibility problems were evidenced for 2 QMs, due to a lack of specific information in medical records. They are indicated in Table 2. For example, this occurred in the QM 'Surgery intervention in patients who were symptomatic after a minimum of 6–12 months of conservative therapies’ because pain assessment was not measurable on some patients. Reliability indexes are displayed in Table 2 for each potential indicator.

All indexes indicated substantial to excellent agreement.

### Supporting evidence

Brief descriptions of the literature that supports the criterion of quality for each of the indicators are provided below.

### Patient examination

#### Physical examination

Patients with cervical radiculopathy, proximal neurovascular entrapment and radial tunnel syndrome [2] may complain of the same symptoms as patients with LE. However, there is no “gold standard” for the diagnosis of LE, and orthopaedic tests such as pain with resisted wrist extension (Cozen’s sign) are traditionally recommended for differential diagnosis [22].

#### *Pain and functional assessment*

The literature review failed to identify clinical studies that evaluated relations between outcomes and assessment of pain or functional limitations. However, because improvement of pain and function are two primary goals in the treatment of LE [23], it seems that these parameters are essential for clinical decision making.

### Educational interventions

A variety of factors, such as force, repetition, posture [24-26] and specific combined elbow exposure, such as combined elbow flexion/extension, wrist bending and perceived physical exertion may lead to LE [27,28]. These factors are not only risk factors for developing LE, but also indicators of poor prognosis and prevention [29]. For prevention, patient education is important to reduce the ergonomic risk [29].

#### *Effectiveness of patient education*

The literature search identified 3 published studies for the effectiveness of patient education on pain and disability among individuals with LE [28-30], all of which shared similar conclusions. The most recent paper [30] concluded that a structured physiotherapy treatment programme, that included ergonomic advice, was more effective than corticosteroid injections and NSAIDs, the major findings being that the intervention group had less pain than patients treated with corticosteroid injections or NSAIDs and experienced better function than those treated with corticosteroid injections. Furthermore, the intervention group had a lower recurrence and fewer sick leave days.

### Pharmacological therapy

#### *First line*

Non-steroidal anti-inflammatory drugs (NSAIDs) via oral administration have been used extensively for many years to treat pain associated with LE. There is some evidence for a short term benefit of NSAIDs (2 weeks) with a decrease of pain and function, but this benefit was not sustained [31,32]. However, there is little evidence to support the use of oral NSAIDs in the long term.

#### *Prophylaxis of gastrointestinal bleedings (GI)*

Numerous RCTs and meta-analyses have demonstrated that NSAIDs are associated with a greater risk of GI bleedings. Exposure to NSAIDs has been associated with a 2.2 to 5.4 greater risk of various adverse GI events [33]. A variety of factors such as older age (age ≥75), peptic ulcer disease, history of adverse GI events, and concomitant therapy with anticoagulants or corticosteroids, may exacerbate the NSAID-associated risk for GI toxicity [34]. One meta-analysis of 112 RCTs [35] found that gastroprotective strategies such as proton pump inhibitor (PPI) reduce the risk of symptomatic ulcers, and misoprostol reduces the risk of serious GI complications.

### Physical therapy

#### *First line*

**Therapeutic exercises** Numerous RCTs have evaluated the effects of exercise on LE. Four SRs [24,25,36-38] reached similar conclusions. The most recent SR [36] evaluated the effect of different exercises in LE on pain and disability. Of the 12 included studies, 9 addressed the effects of isotonic (eccentric/concentric) exercises, 2 studied the effect of isometrics and one studied isokinetic exercises. All studies reported that resistance exercises resulted in substantial improvement in pain and grip strength.

The most recent RCT evaluated the short-term effects of daily eccentric exercises on functional pain-free hand strength in subjects with long-term LE [25]. The exercise program included 2×8–12 repetitions once a day during the first week, while the instruction for the following 2 weeks was to progress to twice daily. At the end of the intervention, the exercise group had significantly higher pain-free hand-grip strength and higher pain-free hand-extensor strength, and in the exercise group the proportion of cases with LE decreased by 66% at the end of the intervention, whereas in controls they decreased by 21%.

**Manual therapy** One SR [26] showed that lateral-glide mobilization with movement technique had positive effects for pain relief and restoration of function in patients with LE. One RCT [39] studied the effect of applied mobilization techniques using a program of six repetitions performed with a 15 second rest interval between repetitions. This study demonstrated a significant and substantial increase in painfree grip strength of 58% (of the order of 60 Newton) in the treatment group but not in the placebo or control.

**Laser therapy** The most recent meta-analysis [40] assessed the clinical effectiveness of Low Level Laser Therapy (LLLT), the relevance of irradiation parameters to outcomes, and the validity of current dosage recommendations for the treatment of tendinopathy. The review included 25 relevant studies, 13 investigated the effectiveness of LLLT for LE of which 6 showed positive results. As summarized, the positive results evidenced a recommended dosage for the management of LE which was a wavelength of 904 nm and power densities that lay between 2-100 mW/cm^2^.

### Time of referral

NSAIDs are usually prescribed for 2 weeks [31,32], and corticosteroid injections [41,42] are often used if treatment by oral medication and other non-operative interventions have failed. Often, physical therapy is a first option to referral when they are not effective. There is no available evidence of an adequate time of referral after oral NSAIDs; however, the evidence to-date suggests that early multimodal programmes of physical therapy should be recommended after corticosteroid injections.

A RCT [43] supported that the combined approach is preferred to that of injection alone. This study demonstrated that a physical therapy program 1–2 weeks following injection comprising education, 8 sessions of manual therapy techniques (Mobilization With Movement), concentric-eccentric exercises and active home exercises improved the long term efficacy and reduced the recurrence rates. The, benefits gained by adding physiotherapy to injection outweighed the costs associated with injection alone, furthermore the cost-effectiveness of the combined therapy was superior to the cost-effectiveness of injection alone.

### Other therapeutic interventions

#### *Platelet-rich plasma (PRP)*

There is a growing body of supporting evidence for this conservative approach, mainly for patients with LE with refractory symptoms after physical therapy management. Four SRs [42,44-46] have described the clinical efficacy and risk of adverse events of PRP for treatment of LE. The most recent meta-analysis [46] identified that the effects of PRP injections were statistically superior to placebo. Regarding the injection method, the recommendations were to collect an amount of 25 ml of autologous blood to obtain an average of 3.5 cc of plasma, and it was not deemed necessary to use calcium or thrombin prior activation of platelets. For the injection technique it was recommended to perform the infiltration into the extensor digitorum communis tendon using the peppering technique.

#### *Surgery*

Four SRs [8,47-49] have studied the effectiveness of surgical treatment for LE and they reported similar conclusions. Surgical options (percutaneous, open and arthroscopic techniques) were effective and safe interventions in relieving pain and restoring function in cases where non-operative approaches failed. However, these studies were unable to support the superiority of one surgical procedure over another.

## Discussion

This paper summarizes the development of evidence-based QMs for measuring degree of implementation of best practices recommendations for patients with LE. Over a period of one year, 12 QMs were developed and evaluated by a panel according to methodological requirements based on recommendations from the literature [13,14].

In our literature review we appreciated a considerable availability of systematic reviews including patients with LE [8,24,26-28,31,36,40,41,44-46,49]. However, we found none containing specific clinical practice guidelines or clinical quality indicators of LE care. This finding is surprising when compared with the existing tools for quality assessment of other health conditions, such as diabetes, cardiovascular disease, arthritis or low back pain [50-53]. Thus, to our knowledge, the QMs we show here may provide a first step in filling the relative void of quality assessment for LE care.

Although we were able to find solid evidence to support a link between the processes of care described in some QMs and meaningful patient outcomes, no such evidence exists for others, for example, for QMs related to pain and functional assessment. A possible explanation for this lack of evidence could be because these processes were traditionally assumed to be so essential to care that clinical trials assessing their importance have not been performed [22]. Therefore, although we could not find supporting evidence, the expert panel rated these QMs as valid measures of quality because assessment of these parameters is necessary to direct therapeutic approaches.

Most of the QMs must be measured only for some individuals with LE. For this reason, we constructed many QMs including in the denominator and numerator only individuals with LE who should receive the indicated care. For example, not all patients with LE and NSAIDs should receive prophylaxis of GI bleedings by gastroprotective strategies, only those who are of an older age (age ≥75), with a history of adverse GI events, and concomitant therapy with anticoagulants or corticosteroids. In a similar way, not all patients with NSAIDs should receive a multimodal program of physical therapy within 14 days, only those who received a corticosteroid injection.

The set of QMs we present here involves multidisciplinary interventions. All QMs are under control of the professionals whose performance is evaluated, so that it is possible for them to improve that performance. When using these measures for quality improvement purposes within an institution or facility, a baseline assessment of current practice is recommended to better understand the quality problem and to provide motivation for change. Baseline results should also be used to establish a basis for comparison across institutional units or over time [51,54].

Our study had its strengths and limitations. The main strengths are related to the development process. We decided to develop potential QMs using a standardized, rigorous, approved, evidence-based approach following previously defined recommendations to ensure high methodological quality and maximal clearness of our outcomes [13,14,55]. Furthermore, the QMs were developed by a multidisciplinary panel of experts to guarantee a wide acceptance of the results by institutions and organizations engaged in LE care. Additionally, a pilot study was implemented to assure that measuring performance of health care providers with those QMs is feasible and reliable. The findings obtained should be interpreted in light of the limitations of the study. First, many medical records were lacking specific information regarding the history of adverse GI events, and the use of concomitant therapy with anticoagulants or corticosteroids. Consequently, problems of feasibility arose for measuring prophylaxis and time of referral QMs, and sample size for the reliability study was lower than desirable. Secondly, only one patient received PRP and three had a process for longer than one year. Thus, analyses for reliability of surgery and PRP QMs were not conducted here. Therefore, while these analyses can be performed in upcoming studies, the use of surgery and PRP QMs may warrant caution.

## Conclusions

This is the first study to develop and evaluate QMs for patients with LE.

The QMs we present were developed to assess quality and not to represent guidelines of optimal care. However, as there is no published CPG in the literature to-date, the evidence-based recommendations identified in our review could also be useful for developing a CPG to describe a range of diagnostic and therapeutic processes that might be considered best practices.

## Consent

Written informed consent was obtained from the patient for the publication of this report and any accompanying images.

## Competing interests

The authors declare no competing interests in relation to the content of this article.

## Authors’ contributions

FMi, designed the study; participated in literature search, literature review, carried out intra-rater reliability and wrote the paper. FMe, contributed to the initial study idea, study design, data interpretation, and read and approved the final draft. FV, contributed to initial study idea, study design, coordinated the working group, and carried out inter-rate reliability. All authors read and approved the final manuscript.

## Pre-publication history

The pre-publication history for this paper can be accessed here:

http://www.biomedcentral.com/1471-2474/14/310/prepub
